# A Comparative Evaluation of Dye Removal in Batch and Air-Lift Systems by Commercial and Crude Laccase Immobilized on Biochar

**DOI:** 10.3390/polym18202563

**Published:** 2026-10-17

**Authors:** Anja Antanasković, Milan Milivojević, Zorica Lopičić, Suzana Dimitrijević-Branković, Tamara Vujatović-Velimirov, Nevena Ilić

**Affiliations:** 1 Institute for Technology of Nuclear and Other Mineral Raw Materials, Franchet d’Esperey Boulevard 86, 11000 Belgrade, Serbia; 2 Faculty of Technology and Metallurgy, University of Belgrade, Karnegijeva 4, 11000 Belgrade, Serbia; 3 Military Technical Institute, Ratka Resanovića 1, 11000 Belgrade, Serbia; 4 Inovation Centre of Faculty of Technology and Metallurgy, Karnegijeva 4, 11000 Belgrade, Serbia

**Keywords:** lignocellulosic waste biomass, pyrolysis, acid modification, Brilliant Green, air-lift reactor, environmental impact assessment

## Abstract

Biochar has emerged as a highly attractive, sustainable support for enzyme immobilization, with dual benefits: valorization of available lignocellulosic waste and use as a stable, porous matrix for enzyme binding. This study investigated acid-modified sour cherry stone biochar (SCBM) as support for two laccases of different origins: crude laccase (*Bjerkandera adusta* TMF1 (CL)) and purified commercial laccase (*Trametes versicolor* (TvL)). The aim was to compare immobilization efficiency, activity retention and Brilliant Green (BG) decolorization in batch and air-lift reactors (ALRs). Immobilization of crude (SCBM-CL) and commercial (SCBM-TvL) laccase was successfully achieved and confirmed by SEM-EDX and FTIR-ATR analyses. Under optimized batch conditions, SCBM-TvL achieved approximately 92% of BG removal within 4 h and maintained high catalytic activity over nine consecutive cycles, whereas SCBM-CL reached almost 66% removal, with a progressive loss of activity upon reuse. In the ALR, using a 25-fold lower biochar-to-solution ratio, SCBM-TvL achieved almost complete BG decolorization within 4 h, while SCBM-CL achieved 57% within the same time. After treatment, BG solution exhibited a phytostimulatory effect, no antimicrobial activity, and 87% COD reduction, indicating reduced toxicity and organic load after enzyme treatment. Furthermore, the BG decolorization achieved by SCBM-TvL in ALRs highlights the economic advantage of this system and supports its potential for further scale-up.

## 1. Introduction

Fungal laccases have attracted increasing attention in recent decades, particularly laccases derived from white-rot fungi. Broad substrate specificity and high catalytic efficiency make fungal laccases promising biocatalysts for a wide range of industrial and environmental applications. Among their various applications, the ability of fungal laccases to oxidize a broad spectrum of organic compounds has particularly stimulated interest in their use for dye decolorization. Laccases catalyze the one-electron oxidation of phenolic and other aromatic compounds, generating reactive radical intermediates that undergo subsequent transformations and contribute to dye decolorization [1,2]. Laccases from different fungal sources may vary considerably in their degree of glycosylation, molecular size, and kinetic properties, which can affect their catalytic performance and stability. In addition, laccase can be obtained as a crude extract or in purified (commercial) form, resulting in differences in biochemical characteristics and applicability. Purified laccases typically exhibit higher specific activity and well-defined kinetic parameters due to the removal of non-enzymatic components during downstream processing, allowing more reproducible and controlled reactions. In contrast, crude enzyme extracts may contain natural mediators and stabilizing compounds that can enhance enzyme performance and offer a more cost-effective alternative, thereby increasing their potential for large-scale industrial applications [3,4]. The lower cost of crude laccase may be crucial for environmental processes which are often non-profit, and therefore lower operational cost is of the utmost importance.

Despite these advantages, the application of laccases in free form is often limited by their relatively low operational stability, difficulties in enzyme recovery, and loss of activity during repeated use [4]. To overcome these limitations and improve the practical applicability of laccases, immobilization on suitable solid supports offers a promising approach. Optimization of the operational parameters of immobilization, particularly pH and temperature, together with the utilization of enzymatic reactors and enzyme reusability, should be considered as important factors for achieving high process efficiency [5]. For efficient enzyme immobilization, the carrier should retain the catalytic activity of the enzyme while providing stability, limiting enzyme leaching and nonspecific adsorption, allowing regeneration, and remaining economically viable [6,7]. Moreover, growing attention has been directed toward the development of environmentally friendly and sustainable materials as supports for enzyme immobilization. Possessing many of the desirable characteristics of an ideal immobilization support, biochar has emerged in recent years as a promising material for enzyme immobilization. Owing to its high surface area, porous structure, stability, and abundance of surface functional groups, biochar represents a promising support for enzyme immobilization [6,8]. A recent study by Zhang et al. [9] showed that research on enzymes immobilized on biochar for environmental remediation has increased in recent years, with the highest number of publications reported in 2023. For example, Naghdi et al. [10] demonstrated that laccase immobilized on acid-treated pinewood nanobiochar efficiently removed 83% of carbamazepine from spiked water. Similarly, Rasheed et al. [11] developed NH_2_-functionalized magnetic biochar from spent mushroom substrate for laccase immobilization, achieving more than 90% removal of aflatoxin B1 within 5 h. However, all of these studies were conducted in batch systems, which are simple and convenient for laboratory-scale investigations. For the scale-up application of immobilized enzymes, their performance should also be assessed in reactors.

An air-lift reactor (ALR) combines a simple design with flexible operation by utilizing a gas stream, typically air, which serves simultaneously for aeration and mixing while driving controlled liquid circulation. The introduction of compressed air enables simultaneous gas–liquid–solid phase mixing and liquid recirculation. An ALR offers several operational advantages, including a mechanically simple design, ease of maintenance, high solid-phase content, efficient heat transfer, moderate interphase mass transfer, and effective mixing at relatively low energy consumption [12,13]. Compared with batch systems, ALRs operate under lower shear forces due to the turbulent flow field and directed liquid circulation, resulting in a more uniform distribution of shear forces throughout the reactor column. This characteristic makes ALRs particularly suitable for processes involving biological catalysts, such as enzymes, as the reduced and controllable shear stress may help preserve their stability and catalytic functionality [14,15].

Sour cherry stone lignocellulosic biomass, used as a starting material in present study, is composed mainly of natural polymers, including cellulose, hemicellulose, and lignin, which are transformed during pyrolysis into biochar. The conversion of this agro-industrial waste into a functional enzyme carrier provides an additional value-added application for this polymer-based waste biomass, which is in accordance with the principles of a circular economy. In this study, acid-modified sour cherry stone biochar (SCBM) was investigated as a support for the immobilization of two laccases: crude laccase produced by *Bjerkandera adusta* TMF1 and a purified (commercial) laccase from *Trametes versicolor*. The immobilization process was optimized for both enzymes, and the properties and decolorization efficiency of the resulting biocatalysts were subsequently evaluated using Brilliant Green (BG) as a model dye. To the best of the authors’ knowledge, no previous study has directly compared crude and commercial laccases immobilized on the same biochar support for dye decolorization. The decolorization process was investigated in a batch system, and the immobilized biocatalysts under the established conditions were subsequently evaluated in an air-lift reactor. The novelty of this study lies in the first investigation of acid-modified sour cherry stone biochar as a support for *Bjerkandera adusta* TMF1 laccase, together with a direct comparison of its performance with that of a commercial laccase immobilized on the same support. Most importantly, to the best of the authors’ knowledge, this is the first study to evaluate a biochar-supported enzyme system in an air-lift reactor, thereby introducing a novel reactor configuration for the application of biochar-based biocatalysts and providing a basis for their further development toward scale-up application.

## 2. Materials and Methods

### 2.1. Materials

Sour cherry stones (*Prunus cerasus* L.) were sourced from the local factory “Vino Župa”, where they were generated as agro-industrial waste. The obtained material was further processed to produce biochar (SCB), which was subsequently modified via acid treatment (SCBM) according to the procedure described in our previous study [6].

Commercial laccase from *Trametes versicolor* (powder, ≥0.5 U/mg) was supplied by Sigma-Aldrich, Germany, while crude laccase was produced by the fungal strain *Bjerkandera adusta* TMF1 (accession number MW327505) according to the procedure reported by Ilić et al. [16]. The crude laccase, previously characterized by SDS-PAGE, exhibited a laccase activity of 51.51 IU/mL and a heterogeneous protein profile with a predominant band at approximately 50 kDa, while no MnP or LiP activity was detected [16]. Prior to immobilization, the crude laccase preparation was diluted to an initial laccase activity of 450 IU/L.

ABTS (2,2-azinobis-(3-ethylbenzthiazoline-6-sulfonic acid), used as a substrate for the activity assay of commercial laccase, and Brilliant Green (C_27_H_34_N_2_O_4_S), used as a model pollutant for degradation studies, were purchased from Sigma-Aldrich, St. Louis, MO, USA. All other chemicals were of analytical grade and used as received.

### 2.2. Laccase Immobilization

Commercial laccase from *Trametes versicolor* (TvL) and crude laccase from *Bjerkandera adusta* TMF1 (CL) were immobilized onto modified biochar derived from sour cherry stones (SCBM), and are designated as SCBM-TvL and SCBM-CL, respectively. The success of immobilization was evaluated through residual activity (*RA*) and immobilization efficiency (*IE*). The effects of key operational parameters, including pH (4–8), temperature (20–50 °C), and immobilization time (1–6 h), were investigated to assess the influence of the investigated parameters on enzyme immobilization, considering the nature of the biocatalyst and the characteristics of the support material. For immobilization, 15 mg of SCB-M was suspended in 2 mL Eppendorf tubes with 1 mL of laccase solution (275 IU/L for TvL and 450 IU/L for CL). The mixtures were incubated under agitation at 200 rpm (KS 4000 I, IKA, Staufen, Germany). After the designated immobilization period, the supernatant was separated and analyzed to determine protein concentration using the Bradford assay as well as laccase activity. Subsequently, the biochar with immobilized enzyme was washed with 1 mL of buffer (0.1 M, pH 5) to remove unbound enzymes and then used for enzyme activity determination.

The activity of free and immobilized laccases was determined as described in our previous study [6] by monitoring the oxidation of ABTS in sodium acetate buffer (0.1 M, pH 5) at 30 °C and measuring the absorbance at 420 nm using a Amersham Biosciences Ultrospec 3300 Pro UV/VIS spectrophotometer, while enzyme activities were calculated according to the following equations [1]:

(1) $$\mathrm{Activity}\;\mathrm{of}\;\mathrm{free}\;\mathrm{enzyme}\;\left[\frac{\mathrm{IU}}{\mathrm{ml}}\right]=\frac{{A\;}_{420}\times V_{t}}{t\;\times \;V_{e}\;\times \;\varepsilon }\;\times D$$

(2) $$\mathrm{Activity}\;\mathrm{of}\;\mathrm{free}\;\mathrm{enzyme}\;\left[\frac{\mathrm{IU}}{g}\right]=\mathrm{Activity}\;\mathrm{of}\;\mathrm{free}\;\mathrm{enzyme}\left[\frac{\mathrm{IU}}{\mathrm{ml}}\right]\times \frac{V}{m_{s}}$$

(3) $$\mathrm{Activity}\;\mathrm{of}\;\mathrm{immobilized}\;\mathrm{enzyme}\;\left[\frac{\mathrm{IU}}{g}\right]=\frac{A_{420\;}\times V_{t}}{t\times m_{e}\times \varepsilon }$$

where *A*_420_ represents the absorbance at 420 nm, *V*_t_ is the reaction volume [mL], *D* represents the dilution factor, *t* is the reaction time [min], *V*_e_ is the enzyme volume [mL], *ɛ* is the molar extinction coefficient of ABTS+ (36 mL/(μmol cm)), *V* is the volume employed for laccase extraction [mL], *m*_s_ is the mass of enzyme [g], and *m*_e_ is the quantity of immobilized laccase [g].

The effectiveness of the immobilization process was assessed in terms of immobilization efficiency (*IE*) and residual activity (*RA*), according to the following equations [17]:

(4) $$\mathrm{IE}\;\left[\%\right]=\frac{c_{i}-c_{s}}{c_{i}}\times 100$$

(5) $$\mathrm{RA}\left[\%\right]=\frac{\mathrm{Activity of immmobilized laccase}}{\mathrm{Activity of free laccase}}\times 100$$

where *C*_i_ is free enzyme protein concentration [mg/mL], and *C*_s_ is the protein concentration measured in the washed supernatant [mg/mL].

### 2.3. Biochar Characterization

The biochar yield (*Y*) was calculated as the ratio of the mass of the obtained biochar, to the mass of the feedstock, according to the following equation:

(6) $$Y\;\left[\%\right]=\frac{\mathrm{Mass}\;\mathrm{of}\;\mathrm{biochar}}{\mathrm{Mass}\;\mathrm{of}\;\mathrm{feedstock}}\;\times 100$$

Nitrogen physisorption analysis was performed at 77 K using an Anton Paar NOVA 600 (Anton Paar QuantaTec Inc., Boynton Beach, FL, USA). Prior to adsorption measurements, the samples were degassed under vacuum at room temperature for 1 h, followed by heating to 100 °C and continued degassing for 2 h. The temperature was then increased to 300 °C, and degassing was continued for an additional 12 h. The obtained adsorption data were analyzed using Kaomi for NOVA software (version 3.01). The pore size distribution and surface area were calculated from the adsorption branch of the isotherm using Quenched Solid Density Functional Theory (QSDFT), with N_2_ at 77 K and a carbon kernel assuming mixed slit/cylindrical pore geometry. The fitting error was 0.3%. The Brunauer–Emmet–Teller theory (BET) was used to calculate the specific surface area. Due to the presence of micropores, BET calculation was carried out in a linear range of the curve within a lower relative pressure range (p/p0 from 0.0084 to 0.0434), in accordance with the Rouquerol criteria [18,19].

The surface morphology and elemental distribution of SCBM with immobilized laccases were analyzed using scanning electron microscopy coupled with energy-dispersive X-ray spectroscopy (SEM–EDX) on a JEOL JSM-6610 LV microscope (JEOL Ltd., Tokyo, Japan).

The surface functional groups of biochar before and after laccase adsorption were analyzed using Fourier-transform infrared spectroscopy in attenuated total reflectance mode (FTIR-ATR). The spectra were recorded on a Thermo Nicolet 6700 FTIR spectrometer (International Equipment Trading Ltd., Madison, WI, USA) over the spectral range of 400–4000 cm^−1^.

The point of zero charge (pH_pzc_) of the biochar, before and after modification, was determined by the pH drift method as described in our previous study [20].

### 2.4. pH and Temperature Optimum of Free and Immobilized Laccases on Biochar

The pH and temperature optima of free and immobilized laccases on SCBM were determined using ABTS as the substrate. For pH optimization, enzyme activity was measured in buffer solutions with pH ranging from 3 to 8 at a temperature of 30 °C, while temperature optimum was determined in sodium acetate buffer (0.1 M, pH 5.0) over a temperature range of 20–70 °C. The optimum temperature and pH results are expressed as relative activities, where the maximum observed activity was defined as 100%.

### 2.5. Dye Decolorization

A BG stock solution was prepared by dissolving 1.0 g of BG powder in distilled water to a final volume of 1000 mL, while working solutions were prepared by dilution of the stock solution. The main characteristics of BG were presented in our previous work [13]. Dye decolorization by biochar with immobilized laccases was monitored by measuring the absorbance of the dye at 625 nm using a UV/VIS Ultrospec 3300 Pro spectrophotometer (Amersham Biosciences, Buckinghamshire, UK). The decolorization efficiency (*DE*) was calculated according to the following equation:

(7) $$\mathrm{DE}\;\left[\%\right]=\frac{c_{i}-c_{f}}{c_{i}}\times 100$$

where *C*_i_ and *C_f_* denote the initial and final BG concentrations (mg/L), respectively.

Firstly, dye decolorization was evaluated in a batch system by investigating the effects of process parameters, including temperature (20–50 °C), pH (3–8), decolorization time (1–6 h), and initial dye concentration (10–500 mg/L). The parameters were evaluated using a one-factor-at-a-time (OFAT) approach, in which one parameter was varied while the remaining parameters were kept constant. The condition selected for one parameter was subsequently used in the evaluation of the next parameter. Thus, the selected conditions represent the most favorable experimental conditions identified within the investigated ranges using the applied sequential optimization procedure. For the experiments, 15 mg of biochar with immobilized laccase (15.67 IU/g support for SCBM-CL and 15.58 IU/g support for SCBM-TvL) was added to a 2 mL Eppendorf tube containing 1 mL of dye solution, and the mixture was incubated on an orbital shaker.

Following the batch experiments, dye decolorization was further evaluated in an internal loop air-lift reactor (ALR) under optimized conditions (*T*, pH, and *C*_i_), while the contact time was varied to assess the effect of mixing on dye decolorization using enzyme-immobilized on biochar. The ALR used in this study and the corresponding characteristics are presented in the Supplementary Material (Figure S1 and Table S1). The experiment was performed using 60 mg of biochar with immobilized enzymes in 100 mL of dye solution at air flow rate of 5.5 L/h. Samples (2 mL) without biochar particles were withdrawn from the reactor at determined time intervals, and after absorbance measurements, returned back to the system.

In order to assess the contribution of adsorption to BG decolorisation, laccases immobilized on SCBM were deactivated (incubating the immobilized enzymes on SCBM at 100 °C for 1 h) and further examined under the same investigated conditions.

The reusability of immobilized laccases (SCBM-TvL and SCBM-CL) for BG decolorization was evaluated in both batch and air-lift reactor systems under the selected process conditions. After each cycle, the dye solution was separated and the residual BG concentration was determined spectrophotometrically, while the immobilized laccases on biochar were washed twice with immobilization buffer to remove residual dye. In each subsequent cycle, a BG solution with the same initial concentration was added, and the decolorization process was carried out under identical experimental conditions. The laccase activity in the decolorized reaction supernatants and washing supernatants was also determined after each reuse cycle to assess possible enzyme leaching/desorption from the biochar support during operation and regeneration.

All decolorization experiments were performed in triplicate, and results are expressed as mean ± standard deviation (SD).

### 2.6. Environmental Impact Assessment

After dye decolorization using laccases immobilized on biochar, the quality of the treated wastewater was evaluated.

In order to assess the quality and toxicity of the dye solution after enzymatic treatment, the chemical oxygen demand (COD) was determined before and after decolorization, according to the procedure described in our previous study [21]. Briefly, COD was determined using a Lovibond MD 610 spectrophotometer (Tintometer GmbH, Dortmund, Germany). For each analysis, 2 mL of sample was added to pre-measured digestion vials containing reagents for the appropriate COD concentration range (0–150 mg/L or 0–1500 mg/L). After thermal digestion at 150 °C for 2 h using a Lovibond^®^ RD 125 thermoreactor, the samples were cooled to room temperature and analyzed spectrophotometrically. Deionized water was used as a blank.

The potential toxicity of the BG dye and its degradation products formed after laccases-mediated decolorization was evaluated through antimicrobial and phytotoxicity assays, following the procedure previously described by Ilić and coworkers [17]. Antimicrobial activity was assessed using *Lactobacillus rhamnosus* ATCC 7469, *Candida albicans* ATCC 10259, *Escherichia coli* ATCC 25922, and waste brewer’s yeast *Saccharomyces cerevisiae* as test microorganisms, while phytotoxicity was determined using wheat seeds (*Triticum aestivum*). All experiments were performed in triplicate, and results are expressed as mean ± standard deviation (SD). The data were analyzed using two-way analysis of variance (ANOVA), followed by Tukey’s honestly significant difference (HSD) test for multiple comparisons. Statistical significance was considered at *p* < 0.05.

## 3. Results

### 3.1. Biochar Characterization

Biochar yield depends primarily on the feedstock characteristics and pyrolysis operating conditions and represents an important factor for economic feasibility. The biochar yield for SCB was 26.57%, which is in good agreement with the yield of peach stone biochar (28.1%) produced under the same pyrolysis conditions [22].

A high surface area is essential for effective enzyme loading onto the support matrix [23]. Therefore, the surface area and pore volume of SCB before and after acid modification were determined, and the results are presented in Table 1. Pristine SCB exhibited a specific surface area of 182.00 m^2^/g and a pore volume of 0.0744 cm^3^/g. After acid modification, the specific surface area and pore volume reached 306.89 m^2^/g and 0.1298 cm^3^/g, representing notable increases of 67.81% and 74.46%, respectively. This increase can be attributed to the removal of inorganic impurities and residual ash during acid treatment, which unblocks pores previously generated by the evolution of volatile organic compounds and gases during biomass pyrolysis [4]. These results are consistent with the findings of Naghdi et al. [10], who reported that treatment of pinewood nanobiochar with an H_2_SO_4_/HNO_3_ mixture increased the specific surface area and pore volume by approximately 10% and 5%, respectively. Similarly, Abdoul Magid et al. [24] reported that oxidation of corncob biochar with HNO_3_/H_2_SO_4_ increased the specific surface area by 39.7% and the pore volume by 83.3%. The significant differences in the specific surface area and pore volume obtained in this study compared with those reported in other studies can be attributed to differences in the feedstock type and pyrolysis conditions [24].

The pH_pzc_ value provides important information about the changes in the total net surface charge of the material. At pH above the pH_pzc_, the material surface exhibits a predominantly negative charge, whereas at pH values below the pH_pzc_, the surface is positively charged [13]. In this study, the pH_pzc_ of SCB and SCBM are presented in Table 1. SCB exhibits a pH_pzc_ value of 6.45, which is consistent with the near-neutral pH_pzc_ values commonly reported for biochars produced from fruit stones [20,25]. The pH_pzc_ value of SCBM (3.93) was lower than that of SCB, indicating a more acidic surface character. This decrease may be attributed to the higher abundance of acidic functional groups on the SCBM surface, such as carboxylic groups, after acid treatment. This observation is consistent with the FTIR-ATR analysis discussed in our previous study, which confirmed the presence of oxygen-containing surface functionalities after modification with H_2_SO_4_/HNO_3_ [6].

### 3.2. Laccase Immobilization

**Figure 1:**
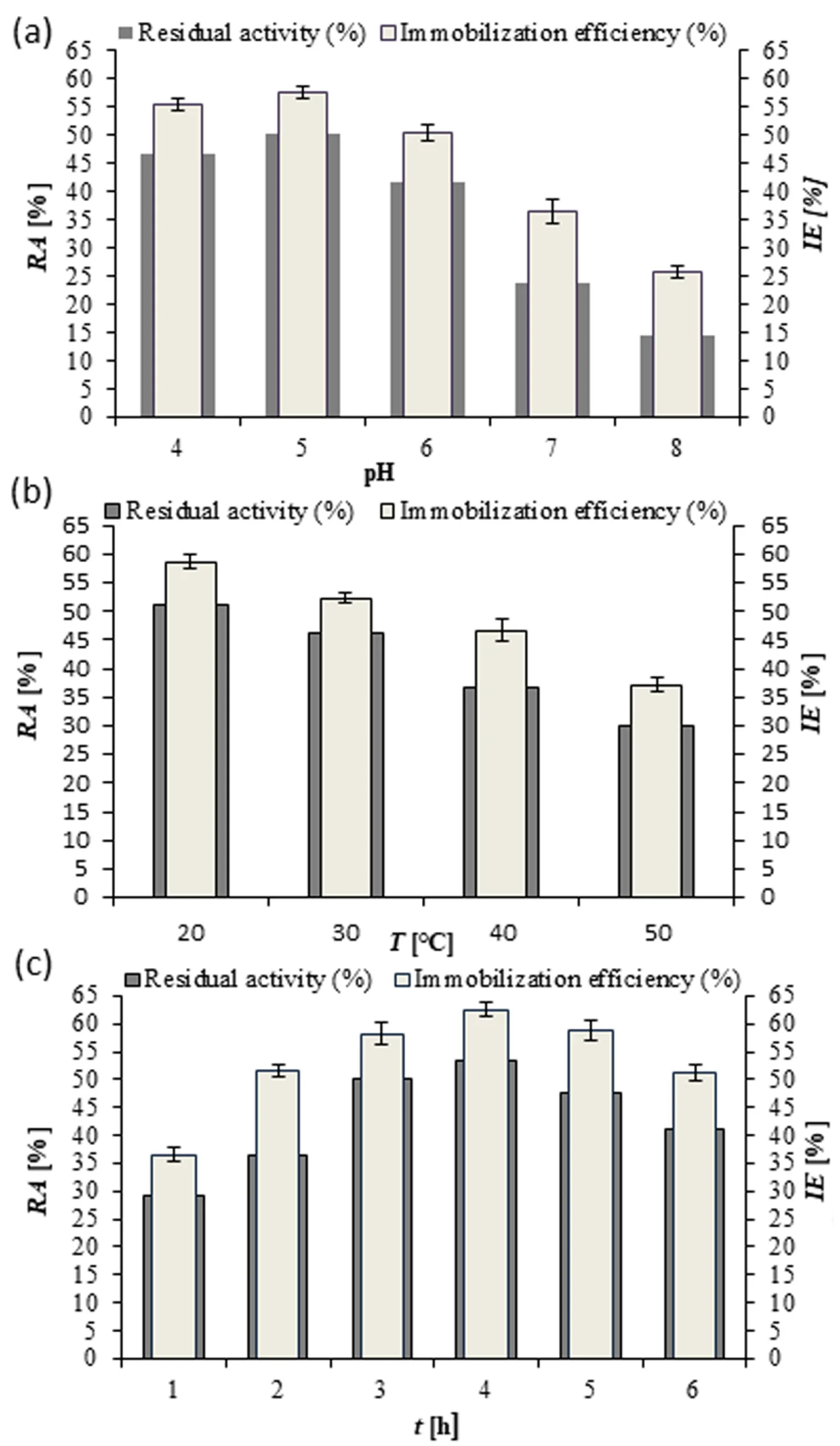
Effect of process parameters, (**a**) pH, (**b**) temperature and (**c**) contact time, on *RA* and *IE* of CL on SCBM.

The immobilization of laccases onto biochar was carried out via the procedure described in our previous paper [6]. In order to maximize enzyme immobilization, the influence of pH, temperature, and contact time was investigated. The influence of these parameters on the immobilization of CL onto SCBM is presented in Figure 1.

The pH plays a crucial role in enzyme immobilization, as variations in pH alter the ionization state of amino acid residues at the enzyme active site and affect enzyme adsorption onto the support. These effects can lead to a decline in catalytic efficiency, reduced reusability, and lower operational stability of the immobilized enzyme system, especially under prolonged operating conditions [6,26]. The effect of pH on CL immobilization onto SCBM was evaluated at room temperature over 3 h, using 15 mg of SCBM per 1 mL of enzyme solution. As shown in Figure 1a, both *RA* and *IE* reached maximum at pH 5, with 50.31% and 57.45%, respectively, indicating that slightly acidic conditions are optimal for the immobilization of CL on SCBM. A further increase in pH led to a gradual decline in both parameters, which dropped to 14.52% for *RA* and 25.54% for *IE* at pH 8. This behavior can be attributed to changes in the ionization state of functional groups on both the enzyme and the biochar surface, which may weaken enzyme–support interactions and consequently reduce catalytic performance. Ilić et al. [27] immobilized the same crude laccase on alkali-treated sawdust, achieving optimal performance at pH 7, with *RA* and *IE* of 57.64% and 72.24% respectively. This shift toward higher pH values further highlights the strong influence of the carrier surface properties on enzyme–support interactions.

Temperature is also an essential parameter in enzyme immobilization, since enzymes are heat-sensitive and can function only within a specific temperature range [28]. Figure 1b illustrates the effect of temperature on CL immobilization onto SCBM in the range of 20–50 °C, at pH 5 for 3 h. The highest *RA* of 51.02% and *IE* of 58.61% were achieved at 20 °C, which may be attributed to an optimal balance between molecular motion and enzyme stability, thereby promoting more effective interactions between CL and biochar. A further increase in temperature resulted in a decline in *RA* and *IE*, probably caused by reduced enzyme activity due to thermal inactivation at elevated temperatures [28]. These results is in agreement with the study by Naghdi et al. [10], who reported the successful immobilization of crude laccase from *T. versicolor* ATCC 20869 onto H_2_SO_4_/HNO_3_-treated nanobiochar at room temperature, achieving an immobilization yield of 26.4%.

Figure 1c presents the effect of incubation time on CL immobilization onto SCBM at pH 5 and 20 °C. The results indicated that *RA* and *IE* gradually increase with contact time reaching maximum of 52.23% and 61.48%, respectively, after 4 h. The relatively high immobilization efficiency observed in the initial period can be attributed to the extensive surface area of SCBM, which was confirmed by BET analysis (Table 1). Prolonged immobilization time led to a slight decrease in both parameters, possibly due to enzyme desorption or multilayer adsorption on the biochar surface, which may limit the accessibility of enzyme active sites [29]. Thus, extending the contact time between SCBM and CL did not lead to further improvements, and 4 h was selected as the most suitable contact time for CL immobilization onto SCBM. A similar trend was reported by Ilić et al. [27], who found that 3.5 h was the optimal adsorption time for immobilization of the same laccase on alkali-treated sawdust, resulting in *RA* of 57.64% and *IE* of 72.24%.

**Figure 2:**
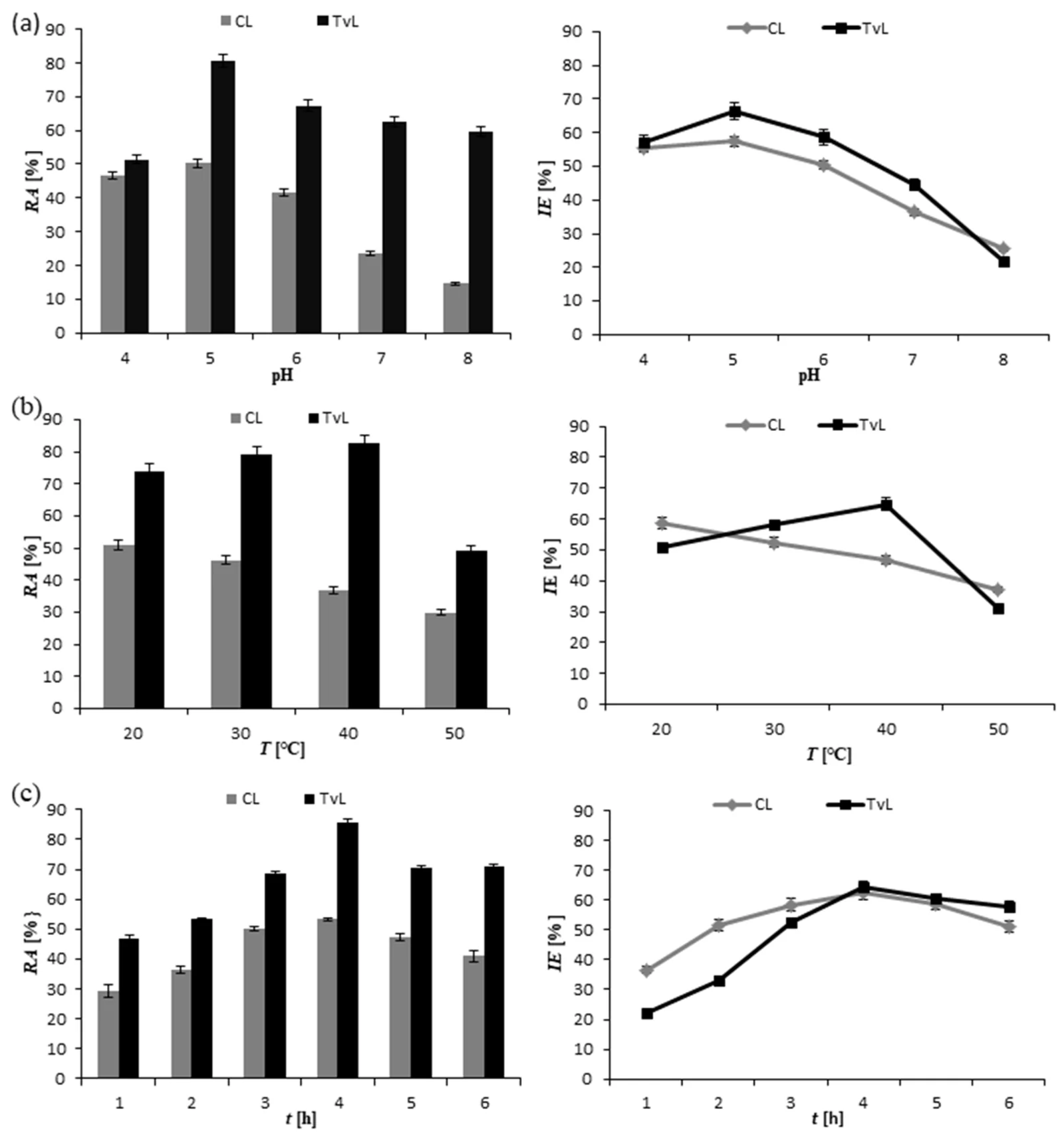
Comparative evaluation of (**a**) pH, (**b**) temperature, and (**c**) contact time on the immobilization of CL and TvL on SCBM.

A detailed investigation of the effects of process parameters on the immobilization of TvL on SCBM has been reported in our previous study [6], while a comparative analysis of CL and TvL is presented in Figure 2.

The same trend of *RA* and *IE* over the investigated pH for both laccases is noted (Figure 2a), with maximum values obtained at pH 5. The identical pH optimum for immobilization observed for both laccases suggests that the immobilization process is strongly influenced by the properties of the carrier [30], which possesses an acidic surface (pH_sus_ = 4.18) as a result of acid treatment. This is in agreement with study by Al-Sareji et al. [28], who determined that pH 5 was optimal for the immobilization of laccase on acid-treated carbon material.

In contrast to pH, the effect of temperature on immobilization onto SCBM (Figure 2b) revealed notable differences between CL and TvL. CL showed the highest *RA* (51.02%) and *IE* (58.61%) at 20 °C, whereas TvL achieved maximum *RA* (82.77%) and *IE* (64.00%) at 40 °C. These differences in temperature dependence suggest distinct thermal behaviors of the two laccases during immobilization, likely due to variations in enzyme robustness at elevated temperatures.

The effect of immobilization time (Figure 2c) showed that both CL and TvL reached maximum *RA* and *IE* after 4 h of immobilization. However, TvL exhibited higher residual activity throughout the immobilization process, while immobilization efficiency during the first 3 h was lower compared to CL. These differences suggest that the two enzymes respond differently during the immobilization process, likely due to the difference in the nature and structure of the enzymes, which play a crucial role in adsorption rate [31].

Overall, SCBM proved to be an effective support for the immobilization of crude and commercial laccase, with maximum *RA* and *IE* values of 52.23% and 61.48% obtained for CL, and 85.43% and 64.00% for TvL under determined immobilization conditions. The higher *RA* and *IE* observed during the immobilization of TvL compared to CL onto SCBM can be attributed to the fact that commercial laccases are purified, whereas crude extracts contain a mixture of metabolites, proteins, and salts that may competitively interfere with enzyme–biochar interactions and reduce immobilization efficiency. Although CL showed lower immobilization performance, it exhibited consistent and predictable behavior, confirming the feasibility of using non-purified fungal laccase preparations for immobilization on biochar. Therefore, both immobilized laccase systems were further evaluated for BG decolorization in batch and air-lift reactors in order to assess the applicability of crude and commercial enzymes under reactor operating conditions.

### 3.3. Characterization of Laccase Immobilized on SCBM

**Figure 3:**
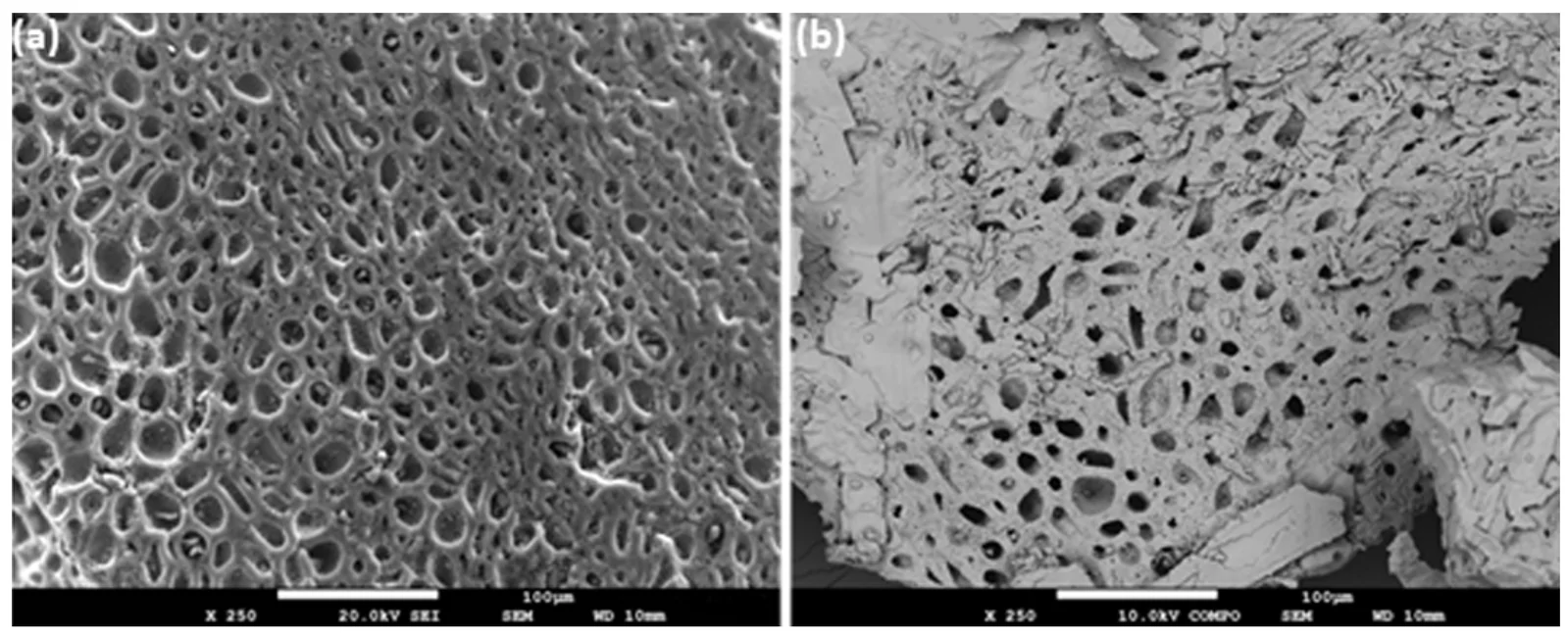
SEM of SCBM: before (**a**) and after CL immobilization (**b**).

SEM images before and after CL immobilization on SCBM are presented in Figure 3. Prior to immobilization, SCBM exhibited a highly developed porous structure with pores of various sizes and irregular distribution, forming a well-defined hierarchical architecture. The presence of pores with different sizes provided a favorable surface for laccase immobilization by offering numerous accessible sites for enzyme attachment [32]. After CL immobilization, the surface appeared smoother and less heterogeneous. This morphological change is likely associated with the deposition of laccase on the biochar surface, leading to partial filling and blockage of the pores. The coating of the rough biochar surface by immobilized laccase molecules may contribute to these observed morphological changes. EDX analysis (Figure S2) provided additional evidence of CL immobilization, as the nitrogen content increased by 11.69 after enzyme loading, which can be attributed to the proteinaceous nature of the immobilized enzyme. These observations are in agreement with the study by Rasheed et al. [11], who reported that laccase immobilized on magnetic biochar reduced surface roughness by filling and coating the porous structure, while EDX and elemental mapping verified protein attachment through a marked increase in nitrogen content.

Compared with TvL immobilized on SCBM, described in our previous study [6], the SEM morphology of SCBM-CL exhibited a more pronounced surface coverage after immobilization, accompanied by partial pore blockage. In contrast to the purified TvL, the crude laccase contains other biomolecules, which likely contributed to the formation of a thicker deposited layer on the biochar surface. Furthermore, EDX analysis (Figure S2) of SCBM-CL revealed the presence of Na, S and Cl, which were not detected in the TvL immobilized system. These elemental signals may be associated with residual components originating from the sodium acetate buffer used for laccase extraction and ammonium sulfate used for enzyme precipitation. The detection of these elements may be related to the crude nature of the enzyme preparation and the possible retention of residual inorganic species during immobilization.

**Figure 4:**
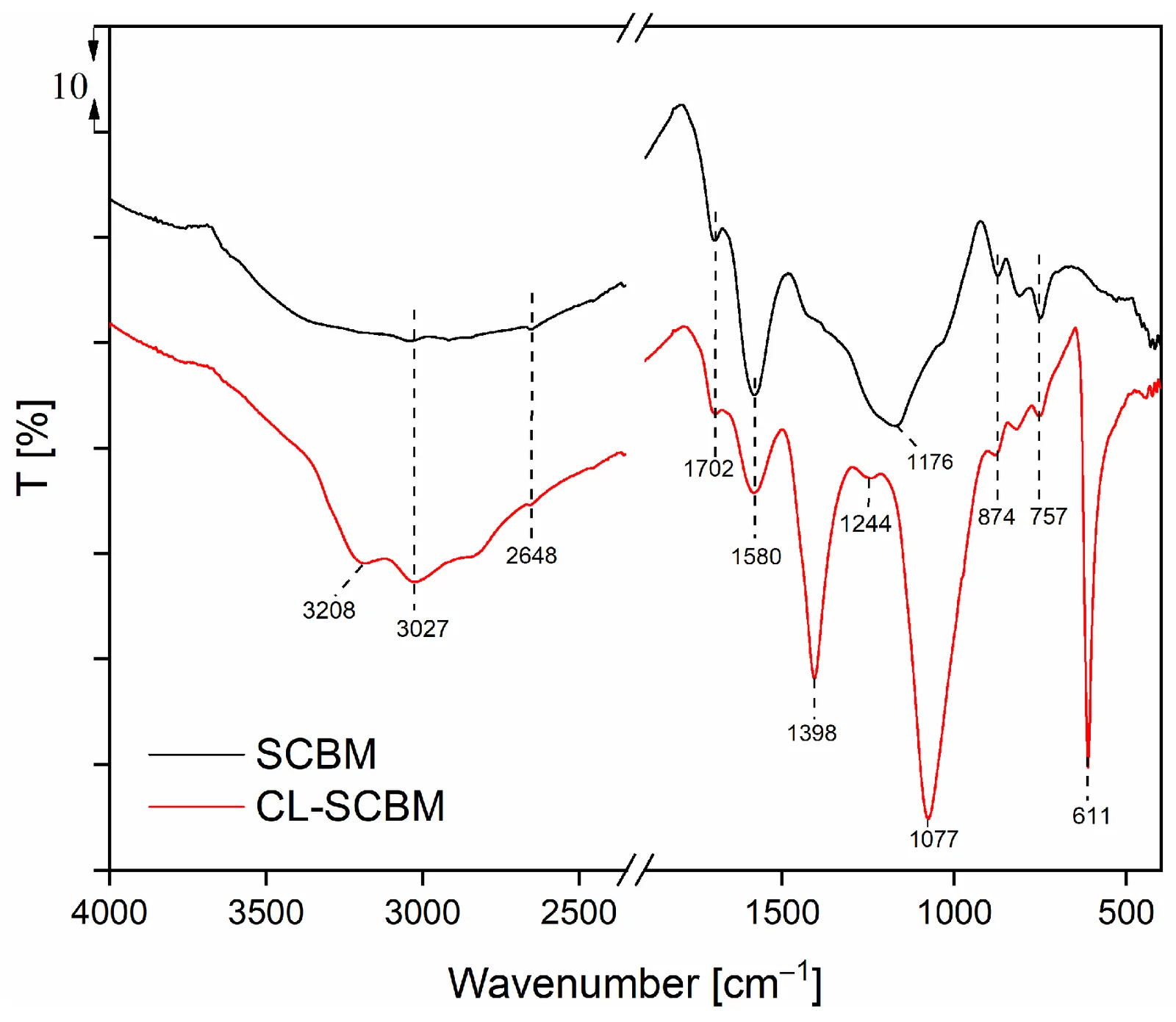
FTIR-ATR spectra of SCBM before and after CL adsorption.

The FTIR-ATR spectra of SCBM before and after TvL immobilization were described in detail in our previous study [6], while the FTIR-ATR analysis of CL immobilized on SCBM is presented in Figure 4. The FTIR spectrum of SCBM revealed the presence of various surface functional groups, including aliphatic and oxygen-containing functional group such as carbonyl and carboxyl, which are characteristic of the biochar structure [6].

Following CL immobilization, a new band at 3208 cm^−1^ appeared, which was assigned to overlapping N–H and O–H stretching vibrations associated with protein and hydroxyl groups [33,34]. The band observed at 1398 cm^−1^ was attributed to C–N stretching vibrations of amine groups [33]. Furthermore, the peak at 1176 cm^−1^, initially assigned to C–O stretching vibrations of phenolic hydroxyl groups in SCBM, shifted to 1244 cm^−1^ after CL immobilization, suggesting the contribution of amide-related vibrations originating from the immobilized enzyme [8,20]. The newly appeared bands at 1077 and 611 cm^−1^ were associated with protein-related vibrations of the laccase structure, further supporting the presence of immobilized enzyme on the SCBM surface [34]. Overall, the FTIR-ATR analysis clearly indicated successful CL immobilization onto SCBM. The *IE* was 61.48% with immobilized laccase activity of 15.67 IU/g support. The appearance of protein-related bands and changes in the surface functional groups confirmed the interaction between laccase and the biochar surface.

### 3.4. pH and Temperature Optimum of Free and Immobilized Laccases

**Figure 5:**
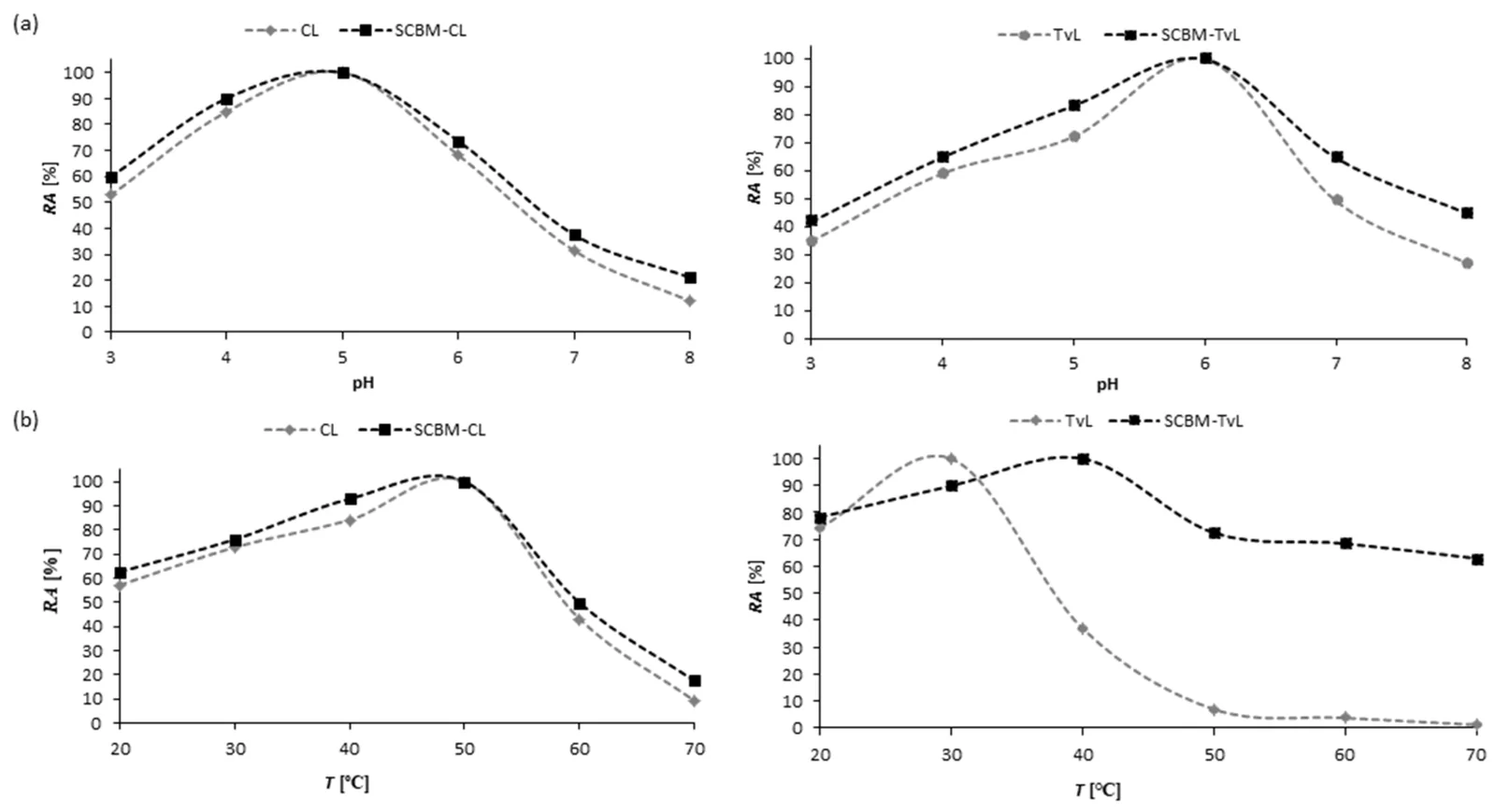
Optimum pH (**a**) and temperature (**b**) of free and immobilized laccases on SCBM.

Critical parameters determining the catalytic activity of laccases include pH and temperature, with the optimal conditions depending on the enzyme source and substrate [32]. In the present study, ABTS was used as the substrate to investigate the effects of pH and temperature on the activity of free (CL and TvL) and immobilized laccases (SCBM-CL and SCBM-TvL). The results are presented in Figure 5.

As shown in Figure 5a, both free and immobilized CL exhibited maximum activity at pH 5. Above the optimum pH, the activity gradually decreased with increasing pH. However, this decrease was less pronounced for SCBM-CL, which retained 21.68% of its relative activity at pH 8, compared with only 10% for free CL. A similar trend was observed for free and immobilized TvL with pH optimum at 6. With a further increase in pH, the activity gradually declined, reaching 27.11% and 44.85% at pH 8 for free and immobilized TvL, respectively. SCBM-TvL retained nearly half of maximum activity under alkaline conditions (pH 8.0), representing a significant enhancement compared to free TvL, indicating that SCBM improved TvL stability by providing a more favorable microenvironment under alkaline conditions. Park et al. [35] also reported that pH 6 is optimal for free and immobilized *T. versicolor* on carbon nanomaterials. The higher relative activity observed for TvL compared with CL after immobilization on SCBM may indicate greater tolerance of this laccase to alkaline conditions. The obtained results are in agreement with previous studies by Borges et al. [36], who reported that fungal laccases generally exhibit their highest activity under acidic conditions, typically within the pH range of 3.0–5.0, followed by a pronounced decrease in activity at neutral and mildly alkaline pH values. This reduction in activity has been attributed to the inhibitory effect of hydroxide ions (OH^−^), which can interact with the copper centers involved in the catalytic process (CuT1 and CuT2). Such interactions may interfere with the electron-transfer pathway from CuT1 to CuT2 and CuT3, thereby impairing the catalytic function of the enzyme [27,36]. The relatively high activity of free and immobilized TvL at near-neutral pH is particularly advantageous, as laccases that retain high activity under neutral or alkaline conditions are highly desirable for specific industrial applications [37]. For example, Colomera and Kuilder [38] reported that the application of alkaline-active fungal laccases (pH > 7) for indigo dye degradation can minimize background staining of denim, thereby offering advantages over conventional acidic treatments and potentially improving the quality and added value of the treated products.

Figure 5b illustrates the effect of temperature on the activity of free and immobilized laccases on SCBM. Free and immobilized CL exhibited a similar trend, with laccase activity increasing progressively from 20 to 50 °C, with the highest activity observed at 50 °C. Further increases in temperature resulted in a gradual decrease in activity, with a pronounced loss observed at 70 °C. This result is consistent with the findings of Fithri et al. [39], who reported an optimum temperature of 50 °C for three fungal laccases isolated from oil palm empty fruit bunches. In contrast, a distinctly different temperature profile was observed for TvL. Free TvL exhibited maximum activity at 30 °C, in agreement with the findings of Ramayanam [40], who also reported 30 °C as the optimum temperature for laccase from *T*. *versicolor*. A sharp decrease in activity occurred with increasing temperature, particularly between 30 and 40 °C, and the enzyme retained only 1.23% of its maximum activity at 70 °C, indicating pronounced thermal inactivation. However, after immobilization on SCBM, the temperature optimum of TvL shifted from 30 to 40 °C. Moreover, although the activity of SCBM-TvL gradually decreased at temperatures above 40 °C, the immobilized enzyme retained 62.98% of its maximum activity even at 70 °C. These findings suggest that immobilization enhanced the thermal tolerance of TvL, helping to shield the enzyme from adverse environmental conditions and thereby expanding its potential for broader applications [41,42].

The different behavior observed for CL and TvL, despite the use of the same SCBM support, may reflect differences in the interaction of these enzymes with the SCBM surface and porous structure. Immobilization can restrict conformational mobility and provide a stabilizing microenvironment, thereby reducing temperature-induced structural changes and thermal deactivation [42]. The extent of this stabilizing effect is likely to depend on the characteristics of the individual enzyme. Thus, the unchanged temperature optimum of CL suggests that its interaction with SCBM did not substantially alter its catalytic behavior, whereas the shift in the temperature optimum and the greater activity at elevated temperatures observed for TvL suggest that immobilization may have improved the conformational stability of the enzyme [27,42]. The porous structure of SCBM may further contribute to this effect by providing physical confinement and limiting conformational changes in the immobilized enzyme under thermal stress [32].

### 3.5. Dye Decolorization

#### 3.5.1. Dye Decolorization in Batch System

**Figure 6:**
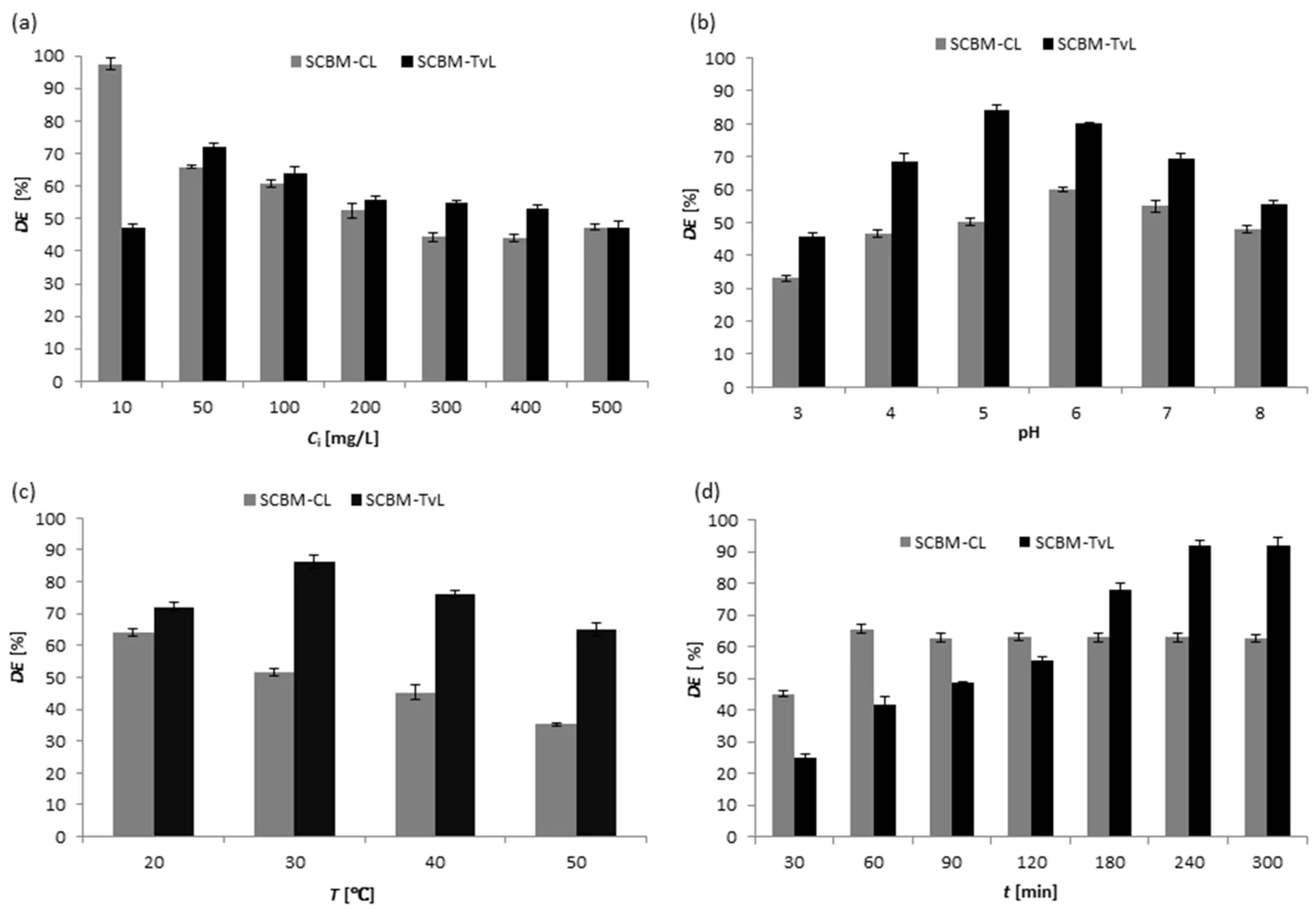
Comparison of the effects of operational parameters on decolorization efficiency using SCBM-CL and SCBM-TvL: (**a**) initial dye concentration, (**b**) pH, (**c**) temperature, and (**d**) time.

Following the successful immobilization of TvL and CL on SCBM, the catalytic activity of the obtained biocatalytic systems was evaluated for dye decolorization. Comparison of SCBM-TvL and SCBM-CL was performed to assess how the different enzyme characteristics affect the *DE* of the resulting biocatalytic systems. Although TvL and CL differ in purity and biochemical composition, the observed differences in decolorization reflect differences between the respective immobilized biocatalytic systems rather than effects attributable solely to enzyme origin. For comparison, the activities of the immobilized biocatalysts were expressed per gram of support, providing a common basis for evaluating decolorization performance, with activities of 15.58 IU/g support for SCBM-TvL and 15.67 IU/g support for SCBM-CL. The comparative results of *DE* obtained using SCBM-TvL and SCBM-CL are presented in Figure 6.

The influence of initial dye concentration on the *DE* of BG by SCBM-CL and SCBM-TvL is presented in Figure 6a. The experiments were performed using different initial dye concentrations ranging from 10 to 500 mg/L, at 20 °C, pH 6 and an incubation time of 3 h. The SCBM-CL system exhibited the maximum *DE* of 62% at initial BG concentration of 10 mg/L, while further increases in dye concentration resulted in a gradual decline in *DE*. In contrast, increasing the initial BG concentration from 10 to 50 mg/L enhanced the *DE* of SCBM-TvL, reaching a maximum *DE* of 75%. However, a further increase in dye concentration led to a decrease in *DE*, similarly to the SCBM-CL system. This behavior may be attributed to substrate inhibition of laccase activity at excessive dye concentrations, as well as the limited availability of accessible catalytic sites for the oxidation of dye molecules [43]. Although a high *DE* was achieved at an initial concentration of 10 mg/L with SCBM-CL, this initial concentration was considered unsuitable for further evaluation due to the low color intensity and the difficulty in accurately monitoring the decolorization process. Therefore, 50 mg/L was selected as the initial BG concentration for subsequent experiments with both immobilized enzyme systems.

The pH of the medium plays a crucial role in enzymatic dye decolorization, as it influences both the ionization state of the enzyme and the charge of the dye molecules, thereby affecting the strength of their interactions. Extremely acidic or alkaline conditions may inhibit enzymatic activity by altering the enzyme conformation and reducing catalytic efficiency [44]. The effect of pH on *DE* of BG by SCBM-CL and SCBM-TvL is presented in Figure 6b. The experiments were conducted within the pH range of 3–8, using an initial BG concentration of 50 mg/L and a contact time of 3 h at room temperature. Both enzymatic systems exhibited a similar trend, with the *DE* of BG increasing until reaching the optimal pH value, followed by a decrease at higher pH. SCBM-CL achieved a maximum *DE* of 62% at pH 6, whereas SCBM-TvL exhibited a markedly higher efficiency, reaching 85% at pH 5. The obtained results are consistent with previous studies reporting that optimal pH for dye decolorization by fungal laccases is generally achieved under neutral to mildly acidic conditions [45].

Temperature significantly influences enzymatic dye decolorization by affecting enzyme activity and stability. Therefore, investigating the effect of temperature is essential for maximizing the catalytic activity of the immobilized enzyme systems [44]. The effect of temperature on *DE* by SCBM-CL and SCBM-TvL was investigated in the range of 20–50 °C, under the following conditions: initial BG concentration of 50 mg/L, contact time of 3 h, and previously selected pH for each enzyme system. The obtained results (Figure 6c) showed that SCBM-CL exhibited the highest *DE* of 64% at 20 °C. In contrast, SCBM-TvL demonstrated higher *DE* across the entire investigated temperature range, with the maximum *DE* of 86.23% at 30 °C. A further temperature increase led to a decline in *DE*, which can be attributed to thermal denaturation of the enzyme and the loss of catalytic activity [46].

The effect of contact time on the *DE* of BG (50 mg/L) by SCBM-CL and SCBM-TvL was monitored over 5 h under the pH and temperature previously determined for each enzyme system. As can be seen from Figure 6d, SCBM-CL exhibited a faster initial decolorization rate, achieving *DE* of 45% after only 30 min, whereas SCBM-TvL showed a lower initial *DE* of 25% for the same time. After 60 min, SCBM-CL reached maximum *DE* of 65.66%, indicating rapid dye decolorization during the initial stage of the process. This is in agreement with findings by Ilić et al. [27], who reported that the same enzyme, immobilized on alkali-treated beech sawdust, achieved *DE* of 63% for mixed dye solution within 30 min. In contrast, the *DE* of SCBM-TvL continuously increased with prolonged contact time, reaching a maximum of 92.11% after 240 min. The slower initial decolorization observed for SCBM-TvL may be associated with differences in the catalytic properties of the enzyme preparations and enzyme–substrate interactions, which can influence the rate of dye oxidation. Li et al. [47] reported that even laccases from the same source may exhibit different substrate specificities toward dye molecules, resulting in variations in their decolorization efficiency.

When using immobilized enzyme systems in decolorization studies, evaluation of contribution of the adsorption (by the carrier) is important, as overall dye removal may result from both enzymatic and adsorptive processes. Simultaneous enzymatic catalysis and adsorption can improve pollutant removal in oxidoreductase-based systems under industrial and environmental conditions, maximizing the efficiency of process combinations [5]. In order to assess the contribution of each process in *DE*, experiments using thermally inactivated laccase immobilized on SCBM were conducted. These results are presented in Figure S3. The deactivated SCBM–CL system exhibited approximately 22% dye removal within the first 60 min, whereas the overall *DE* of the SCBM–CL system reached 65% over the same period. Similarly, the deactivated SCBM–TvL system achieved approximately 40% dye removal within 60 min, compared with an overall *DE* of 45% for the SCBM–TvL system. In both deactivated systems, no substantial increase in dye removal was observed after 60 min, suggesting that adsorption on SCBM accounted for a considerable proportion of the initial dye removal and that most of the available adsorption sites were occupied within the first 60 min. The higher BG removal by adsorption observed for deactivated SCBM–TvL compared with deactivated SCBM–CL may be related to differences in the surface coverage of SCBM by the immobilized enzyme. The crude laccase may contain other components that may occupy active sites on SCBM and reduce availability for BG adsorption, whereas commercial TvL has a higher degree of purification. This is in agreement with the SEM/EDX analysis (Figure 3 and Figure S1), which showed more pronounced surface coverage and partial pore blockage following CL immobilization compared with TvL immobilization. Therefore, the total dye removal of 65.66% with SCBM-CL and 92.11% with SCBM-TvL can be attributed both to the catalytic activity of the enzymes and adsorption onto the biochar. However, the present experiments do not allow these contributions to be quantitatively separated. It may also be hypothesized that adsorption may facilitate the mass transfer of BG molecules from the solution to the enzyme active sites on the biochar surface, thereby contributing to more efficient dye removal [5].

The higher total *DE* observed for SCBM-TvL compared to SCBM-CL may be attributed to several factors. In addition to the higher dye removal achieved by adsorption onto SCBM-TvL, a significant contribution may also arise from the immobilized enzyme. Commercial *T. versicolor* laccase is a well-characterized fungal enzyme with wide substrate specificity and high oxidative potential toward aromatic compounds, including synthetic dyes [44]. In contrast, crude laccase preparations may contain other components that can influence enzyme immobilization and limit catalytic accessibility. Moreover, differences in enzyme–support interactions may affect enzyme orientation and the preservation of catalytic activity after immobilization [23]. Having in mind that SCBM-TvL exhibited higher immobilization efficiency and residual activity than SCBM-CL, indicating that a larger fraction of catalytically active enzyme was retained after immobilization. This enhanced preservation of enzyme activity likely contributed to the superior decolorization of BG.

**Figure 7:**
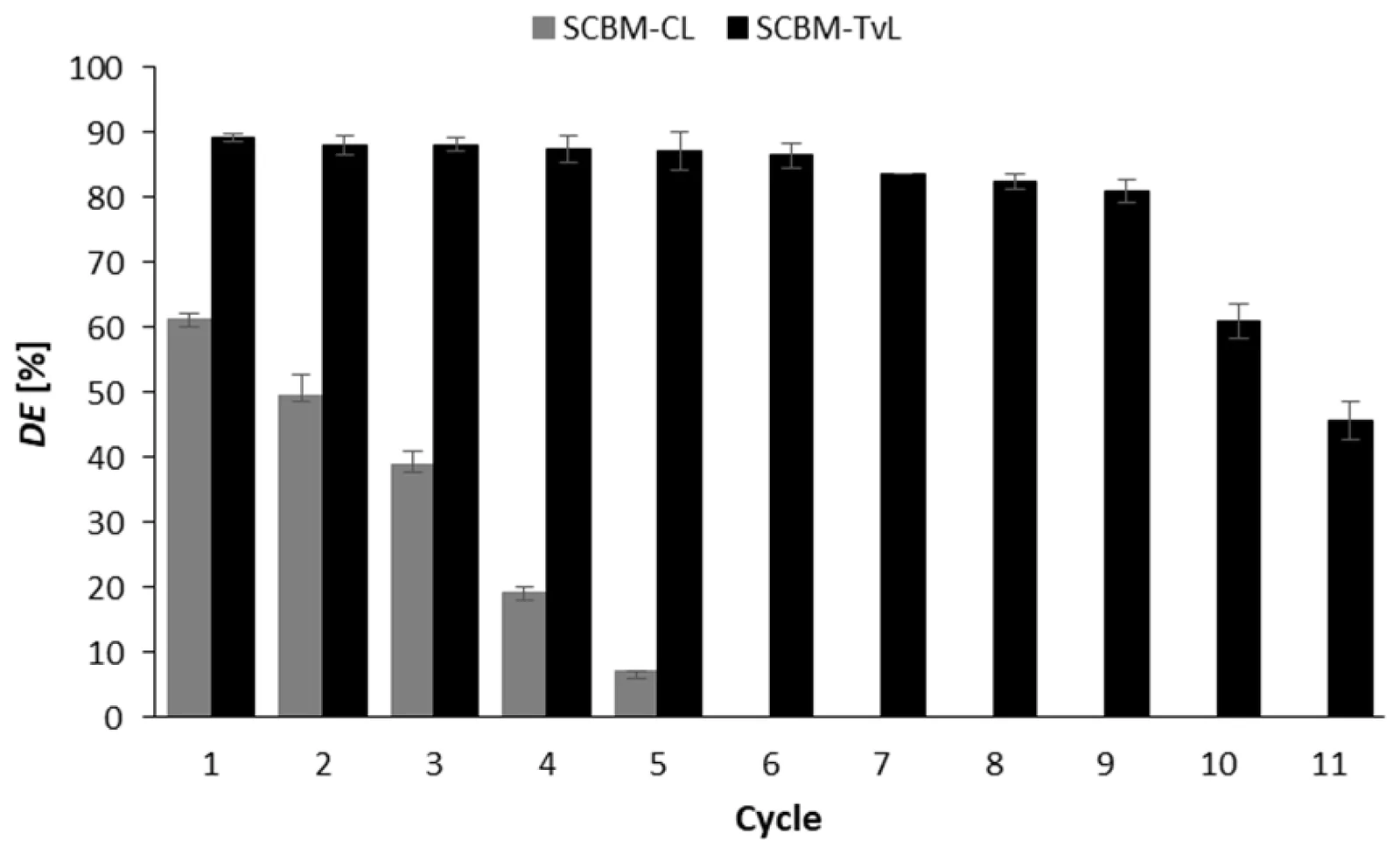
Reusability of SCBM-CL and SCBM-TvL during repeated decolorization cycles.

The reuse of immobilized enzymes is a crucial aspect of industrial biocatalysis, because it significantly influences both economic viability and environmental sustainability. An effective immobilized enzyme should retain significant activity even after repeated cycles [32,46]. The reusability of SCBM-CL and SCBM-TvL for BG decolorization under the previously selected conditions established for each enzyme system is presented in Figure 7.

SCBM-CL was subjected to five consecutive cycles of BG decolorization, during which a progressive decrease in *DE* was observed with increasing cycle number. The *DE* decreased by approximately 11% per cycle, reaching only 8% after five reuse cycles. To further assess the possible contribution of enzyme washout, laccase activity was determined in decolorized reaction supernatants as well as in washout supernatants collected during regeneration after each reuse cycle (Table S2). Laccase activity was detected in the reaction supernatants during all five runs, indicating some extent of enzyme leaching/desorption (approximately 4.21–9.11 IU/L). However, the detected activity represented only a relatively small fraction of the initial immobilized activity. Activity was also detected in supernatants after washing, confirming that part of the enzyme was less bound to the support and could be removed during washing (approximately 1.96–9.56 IU/L). However, the results indicate that enzyme leaching alone cannot explain the pronounced drop in DE. The observed loss of *DE* is therefore likely due to a combination of limited enzyme leaching and other factors, such as gradual deactivation and/or inhibition of the enzyme during reuse. A similar trend was reported by Ilić et al. [16], who applied the same enzyme immobilized onto glutaraldehyde-activated alginate beads for four consecutive cycles of Bromophenol Blue decolorization. In their work, glutaraldehyde was used as a cross-linking agent to facilitate covalent enzyme attachment, whereas in the present study, CL was immobilized onto SCBM solely via adsorption. This demonstrates that biochar can serve as a cost-effective and efficient support for crude laccase and presents a basis for further investigation of immobilization strategies aimed at improving the stability and reusability of the resulting biocatalytic system.

In contrast, SCBM-TvL maintained high *DE* over nine reuse cycles, after which a more significant decline was observed, reaching 45% after the eleventh cycle. Laccase activity was detected in both reaction and wash supernatants during all reuse cycles, with activities in the reaction supernatants ranging from 1.54 to 3.38 IU/L, from 1.96 to 2.99 IU/L in the first wash supernatant, and from 1.21 to 2.44 IU/L in the second wash supernatant (Table S3). The relatively low and stable activities detected in the wash supernatants, particularly during the first nine cycles, indicate that desorption of the enzyme from the SCBM support during repeated use was limited. However, the increase in laccase activity detected in the reaction supernatants during the later cycles, along with the gradual decrease in DE, suggests that enzyme release and/or loss of catalytic activity may have contributed to the decline in decolorization. Alsaiari et al. [48] reported that the same enzyme immobilized on magnetic nanoparticles could be successfully reused for six consecutive cycles for the decolorization of Direct Red 23, while Amari et al. [49] demonstrated that the same laccase immobilized on a magnetic metal–organic support could be applied for five consecutive cycles for the decolorization of Reactive Black 5 and Alizarin Red S dyes. Compared with these studies, SCBM–TvL maintained high overall dye-removal efficiency over a greater number of reuse cycles. Considering the control experiment (Figure S3), in which dye removal by the deactivated SCBM–TvL system approached equilibrium within the first 30 min, the contribution of adsorption is expected to be most pronounced during the first cycle. Therefore, the sustained *DE* observed during subsequent reuse cycles may suggest that the immobilized laccase retained catalytic activity during repeated use. Compared with the findings reported in previous studies [48,49], TvL immobilized on SCBM maintained high dye-removal efficiency over 11 reuse cycles. These results indicate that SCBM represents an efficient and stable support for laccase immobilization, enabling repeated use in dye decolorization processes and highlighting its potential for wastewater treatment applications.

Compared with SCBM-CL, SCBM-TvL exhibited superior reusability, maintaining higher *DE* over a greater number of reuse cycles. These differences suggest that the operational stability of immobilized enzyme systems may depend on several factors, including enzyme characteristic, enzyme–support interactions, and potential enzyme leaching during repeated use [50].

#### 3.5.2. Dye Decolorization in an Air-Lift Reactor

**Figure 8:**
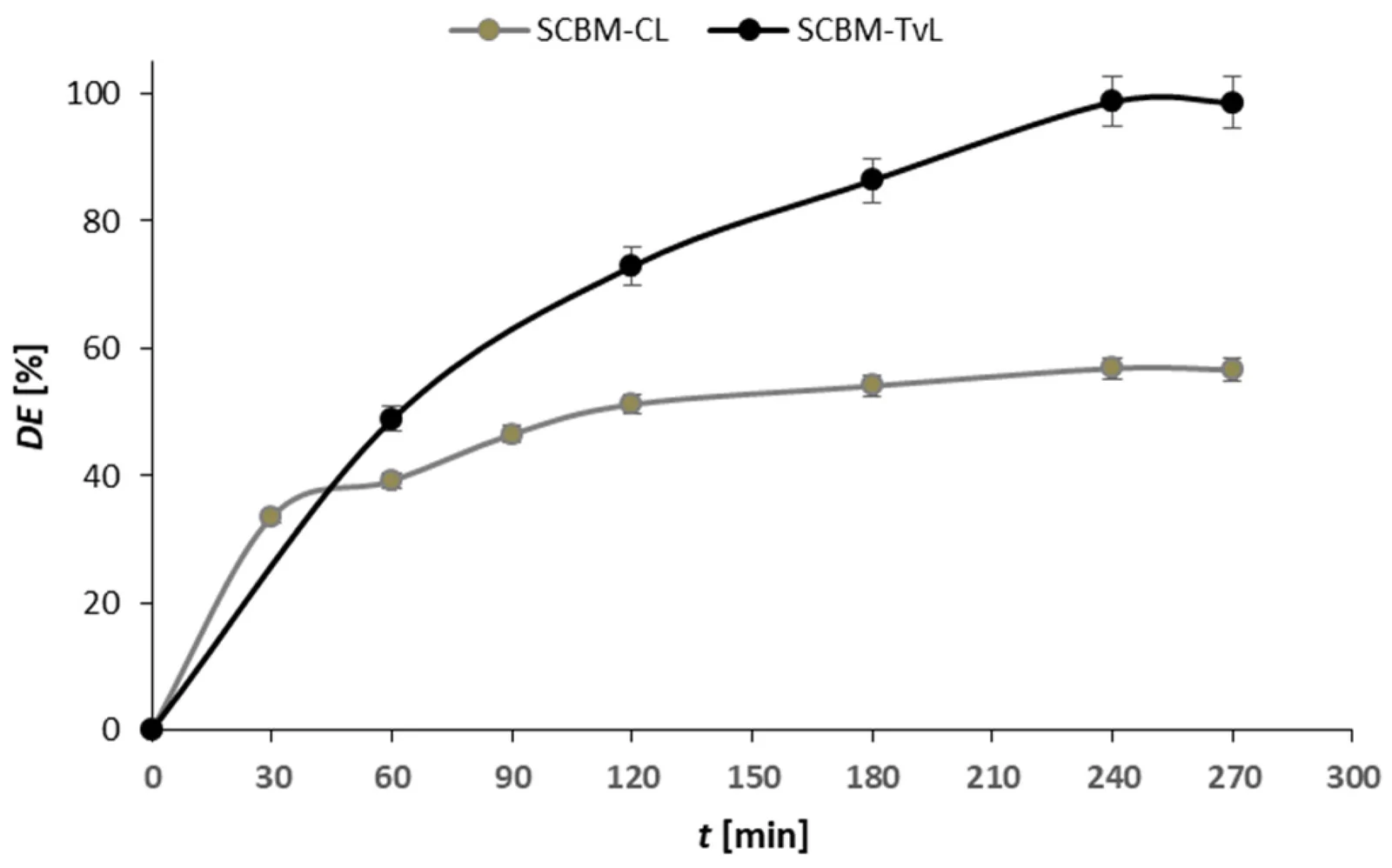
Decolorization efficiency of BG by SCBM-CL and SCBM-TvL in ALR.

Following the optimization of BG decolorization in the batch system, the performance of the immobilized biocatalysts was further investigated in an ALR to evaluate the effect of mixing on enzymes activity. The experiments were conducted under the previously selected pH, temperature, and initial dye concentration for each enzyme system, at a constant air flow rate of 5.5 L/h. Considering the high liquid volume required for operation of the ALR, the biocatalyst dosage was reduced to 0.6 g/L to ensure a more economically feasible process [44]. The total *DE* of BG as a function of time using SCBM-CL and SCBM-TvL is presented in Figure 8, while the effect of adsorption on the overall *DE* is shown in Figure S4.

As shown in Figure 8, SCBM-TvL exhibited higher *DE* compared with SCBM-CL. During the first 60 min, SCBM-TvL removed approximately 50% of BG, whereas SCBM-CL achieved a *DE* of less than 34%. After 240 min, SCBM-TvL almost completely decolorized the dye solution (*DE* of 98%), significantly outperforming SCBM-CL, which reached a maximum *DE* of 57%. The observed *DE* resulted from the combined contributions of BG adsorption and laccase activity. The control results presented in Figure S4 showed that, during the first 120 min, deactivated SCBM-CL adsorbed approximately 18% of BG, whereas deactivated SCBM-TvL achieved approximately 32% BG removal. After 120 min, no further increase in BG removal by adsorption was observed. These results demonstrate that adsorption contributed to the overall BG removal in the ALR, as also observed in the batch system. Consistent with the batch system, the continued superiority of SCBM-TvL indicates that the observed difference is associated with the enzyme origin and its characteristics rather than the reactor configuration.

Notably, the ALR achieved a higher *DE* within the same reaction time using a 25-fold lower biocatalyst dosage compared with the batch system. This improved performance, particularly observed for SCBM-TvL, may be associated with the enhanced oxygen transfer and fluid circulation provided by the air-lift configuration. Aeration is generally reported to facilitate oxygen transfer, which is important for the oxidative catalytic mechanism of laccase, while fluid circulation may help reduce external mass-transfer limitations and facilitate contact between the dye molecules and the immobilized biocatalyst [51]. Although key mass-transfer parameters (such as dissolved oxygen levels and mass-transfer coefficients) were not directly measured in this study, these observations suggest that the ALR configuration offers operational advantages at significantly lower biocatalyst loadings and represents a basis for further research.

**Figure 9:**
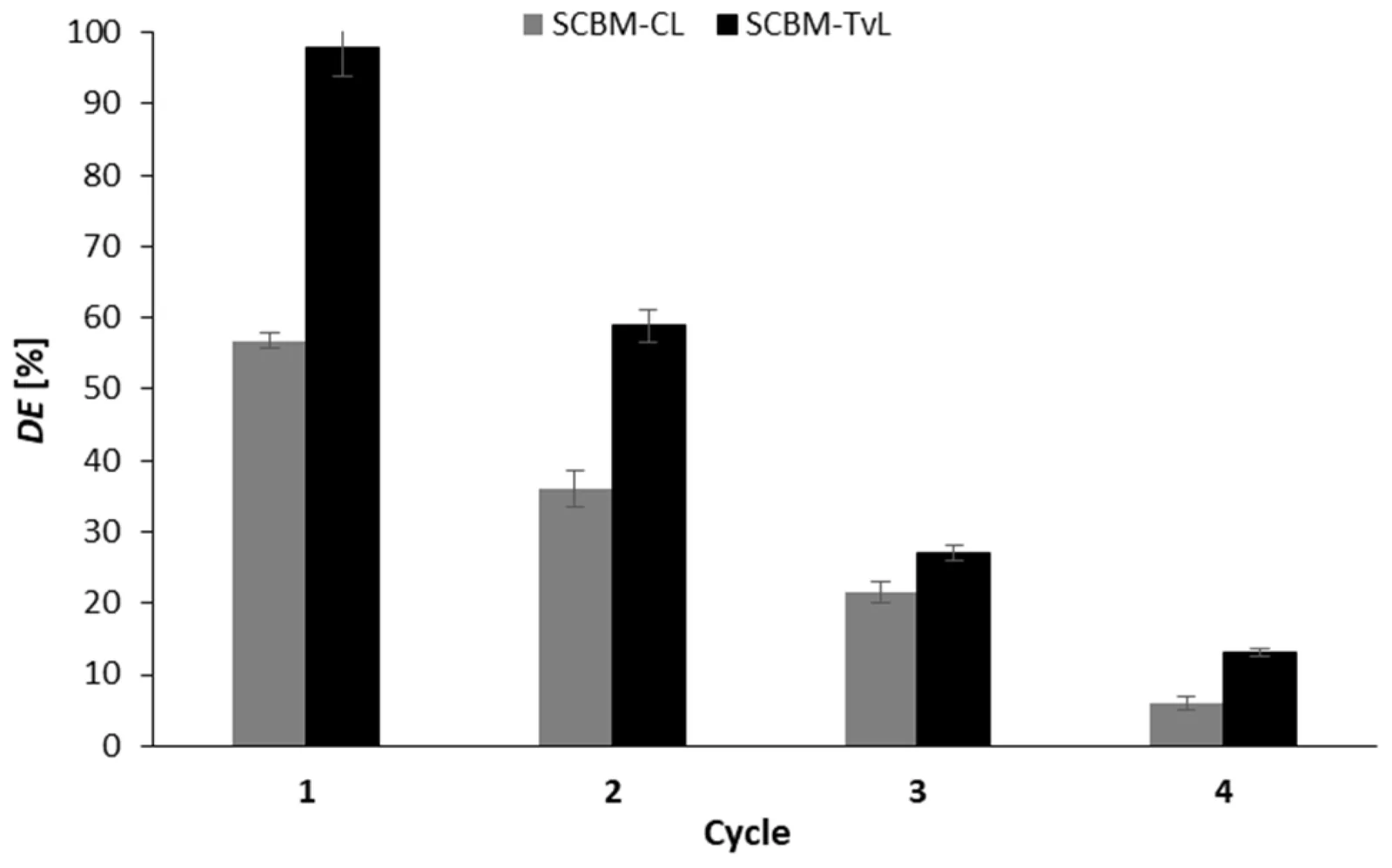
Reusability of SCBM-CL and SCBM-TvL decolorization cycles in ALR.

Figure 9 illustrates the reusability of SCBM-CL and SCBM-TvL for BG decolorization in the ALR. Both biocatalytic systems showed a progressive decrease in *DE* with repeated use. The first reuse cycle resulted in a nearly 40% decrease in *DE* for SCBM-TvL and an approximately 20% decrease for SCBM-CL. This decline became more pronounced with further reuse, with *DE* values reaching only 13% for SCBM-TvL and 6% for SCBM-CL by the fourth cycle. Laccase activity was not detected in either the reaction supernatants or the washing buffers collected after the reuse cycles for either immobilized system, indicating that extensive enzyme leaching from the SCBM support was not evident under the applied ALR operating conditions. Therefore, the pronounced decrease in *DE* is more likely associated with progressive loss of catalytic performance during repeated operation, potentially due to enzyme deactivation and/or inhibition under the applied reaction conditions. The absence of detectable laccase activity in the liquid fractions should nevertheless be interpreted with caution, as any released enzyme may have been present below the detection limit of the activity assay.

The lower reusability observed in the ALR compared to the batch system may be associated with the substantially lower biocatalyst dosage applied (0.6 g/L). At a 25-fold reduced loading, the immobilized enzymes are exposed to a substantially higher substrate-to-enzyme ratio, and the resulting degradation products can lead to enzyme inhibition. Nevertheless, achieving effective BG decolorization under these conditions remains highly noteworthy.

Considering the laboratory-scale nature of the present ALR experiments, the results demonstrate effective BG decolorization at a substantially reduced biocatalyst dosage, particularly in the case of SCBM-TvL. These findings provide a basis for further investigation and optimization of the ALR process, particularly with respect to biocatalyst dosage and air flow rate, as well as improvement of biocatalyst reusability, with the aim of developing a more efficient and economically feasible approach for BG decolorization.

### 3.6. Environmental Impact Assessment

Following the assessment of decolorization efficiency, the environmental impact of the enzymatic treatment was further evaluated by assessing the quality and potential toxicity of the treated solution. Accordingly, COD values were determined in the dye solution before and after enzymatic decolorization by SCBM-CL and SCBM-TvL. Before treatment, the initial BG solution had COD of 872 mg/L, whereas the COD value after treatment with SCBM-CL decreased to 174 mg/L. Furthermore, the COD value of the sample treated with SCBM-TvL decreased to 112 mg/L. These results indicate a substantial improvement in water quality following enzymatic treatment, with COD reductions of 80% and 87% for SCBM-CL and SCBM-TvL, respectively. The pronounced reduction in COD observed for both systems highlights their potential for reducing the organic load of dye-containing wastewater and supports their applicability in real-world wastewater treatment systems.

Phytotoxicity assessment is a crucial in evaluating the environmental safety of synthetic dyes and their transformation products, as it provides insight into their potential impact on early plant development and ecosystem functioning. Seed germination assays are widely employed for this purpose due to their high sensitivity and ecological relevance, since germination represents a key initial stage in plant growth that is particularly vulnerable to toxic compounds [17]. This assessment is particularly important for triphenylmethane dyes, whose persistence and transformation products may adversely affect seed viability and subsequent plant development [21]. The phytotoxicity of BG and its enzymatic degradation products was assessed using the germination index (GI) of *T. aestivum* seeds, calculated based on the relative germination value (RGV) and relative length value (RLV) of *T. aestivum* seeds as described in [27]. According to the established classification, GI values below 65% indicate phytotoxicity, values above this threshold suggest the absence of phytotoxic effects, while GI values exceeding 100% are generally considered indicative of a phytostimulatory response. The results are presented in Table 2.

Untreated BG at concentration of 50 mg/L exhibited pronounced phytotoxic toward wheat seeds, resulting in GI of 38.24%. Following treatment with SCBM-CL, the GI increased substantially to 87.51%, indicating that the degradation products no longer exerted phytotoxic effects on seed germination. An even greater increase was observed after treatment with SCBM-TvL, where the GI reached 116.58%. This value suggests not only complete detoxification of the dye but also a phytostimulatory effect, indicating the potential applicability of the treated solution for agricultural purposes.

A comparable trend has previously been reported for the triphenylmethane dye Malachite Green (MG). The untreated dye exhibited phytotoxicity toward wheat seeds, with a GI of 43.62%, whereas enzymatic decolorization using laccase from *Schizophyllum commune* TMF3 resulted in a pronounced phytostimulatory effect [21]. The similar results observed for BG and MG may be related to the structural similarity of these triphenylmethane dyes. Enzymatic oxidation of these dyes may lead to transformation products with lower phytotoxicity and under certain conditions, may even generate compounds that stimulate seed germination. Overall, these findings demonstrate that laccase-mediated treatment can effectively reduce the phytotoxicity of BG, while treatment with SCBM-TvL showed the most pronounced effect. This suggests that treatment by laccase immobilized on biochar may not only mitigate the phytotoxic effects of triphenylmethane dyes but also generate degradation products with potentially beneficial effects on early plant development.

The antimicrobial activity of BG before and after treatment with SCBM-CL and SCBM-TvL was evaluated, and the results are presented in Table 3. Prior to treatment, BG exhibited an inhibitory effect on tested microorganisms, with inhibition percentages of 25.17% for *E. coli*, 33.67% for *L. rhamnosus*, and 44.12% for *C. albicans*, whereas no inhibitory effect was observed for *S. cerevisiae*. After treatment with SCBM-CL, the inhibitory effect was no longer observed. Instead, a slight stimulation of microbial growth was detected for *E. coli* (7.32%), *L. rhamnosus* (2.70%), and *S. cerevisiae* (9.60%), while no significant effect was observed for *C. albicans*.

Similarly, treatment with SCBM-TvL converted the inhibitory effect of BG into growth stimulation for all tested microorganisms. The observed stimulation reached 13.24% for *E. coli*, 9.13% for *L. rhamnosus*, 8.41% for *C. albicans*, and 14.45% for *S. cerevisiae*. Overall, both enzymatic treatments reduced the inhibitory effect of BG, indicating a reduction in its adverse effects on microbial growth. The higher stimulation values observed after treatment with SCBM-TvL may further indicate a greater reduction in the biological toxicity of BG compared with SCBM-CL.

Statistical analysis revealed significant differences among the tested microorganism–treatment combinations (*p* < 0.05). The results of Tukey’s HSD multiple-comparison test are indicated by different uppercase letters in Table 2, whereas means sharing at least one uppercase letter are not significantly different. Similarly, significant differences among the tested parameters and treatments in the phytotoxicity assay were observed (*p* < 0.05), with the results of Tukey’s HSD multiple-comparison test indicated by different uppercase letters in Table 3. Means sharing at least one uppercase letter are not significantly different.

Overall, both enzymatic treatments reduced the inhibitory effect of BG, indicating a reduction in its adverse effects on microbial growth. This finding is consistent with the study by Vigneshpriya et al. [52], who reported that untreated BG exhibited antimicrobial activity against the tested microorganisms, whereas no inhibition was observed following treatment of the dye with *Sargassum wightii*. The higher stimulation values observed after treatment with SCBM-TvL may further indicate a more effective detoxification of BG and/or formation of metabolites that were less toxic and potentially favorable for microbial growth.

### 3.7. Economic Considerations

Although commercial laccase showed somewhat better results in dye-removal efficiency and reusability, it has to be kept in mind that processes of dye removal are non-profitable and do not require pure enzymes. Therefore, the primary factor that will determine which enzyme will be applied is purely economic. Having in mind that commercial laccase can cost several times more per unit of activity than crude laccase, it is not surprising that some techno-economic studies show that replacing commercial-grade laccase with crude fungal preparations can reduce total treatment and process operational costs by roughly 70% to over 80% [53]. While prices of highly purified, isolated, and formulated-for-stability commercial laccase range broadly from $0.40 to over $150 per kU (kilo-unit of enzymatic activity), depending on purity, source organism, and supplier markup [7], those of crude laccase were estimated as $0.05 to $3.90 per kU depending on scale and processing [54,55]. Therefore, the price of crude laccase, much lower than commercial laccase, makes its use more economically affordable, especially for high volume processes. In addition, the application of ALRs can make those processes even cheaper by reducing energy demands for mixing and due to its smaller carbon footprint [13]. Therefore, despite the better performance of commercial laccase observed in the present study, the substantial economic advantage of crude laccase makes it a more promising candidate for further process development. Accordingly, further studies will focus on the optimization of crude laccase immobilization and stabilization, including cross-linking strategies, as well as its long-term performance in air-lift reactor systems.

## 4. Conclusions

In this study, sour cherry stone biochar was investigated as an effective and sustainable support for the immobilization of both crude and commercial laccases. The valorization of this agricultural waste not only mitigates the environmental burden associated with landfill disposal, but also converts a low-cost polymer-based byproduct into a high-value functional material, contributing to circular economy principles. Acid modification of the biochar significantly enhanced its textural properties, increasing the specific surface area from 182.88 to 306.89 m^2^/g and the total pore volume from 0.0744 to 0.1298 cm^3^/g, thereby providing a more accessible porous framework for enzyme attachment. The modified biochar demonstrated high suitability for both enzymes, achieving maximum residual activity and immobilization efficiency of 52.23% and 61.48% for crude laccase and 85.43% and 64.00% for *T. versicolor* laccase under the selected immobilization conditions. Successful enzyme immobilization onto the porous matrix was confirmed by FTIR-ATR, SEM, and EDX analyses. In batch system, SCBM-TvL demonstrated exceptional operational stability, achieving decolorization efficiency of 92% within 4 h and maintaining high efficiency across nine repetitive cycles, whereas SCBM-CL achieved nearly 66% removal within 2 h, with progressively decreasing in each subsequent cycle. Importantly, unlike previous studies that focused primarily on batch experiments, the performance of biochar-immobilized laccases was additionally investigated in an air-lift reactor. SCBM-TvL achieved nearly complete dye decolorization within 4 h, being applied in ALR with 25-fold lower carrier-to-solution ratio, possibly as a result of enhanced oxygen availability and intensive fluid mixing. On the other hand, SCBM-CL achieved a *DE* of 57% within the same time. Notwithstanding the lower efficiency observed in the ALR, applying the crude enzyme provides a valuable basis for further investigation, particularly regarding enzyme purification and formulation strategies aimed at developing more cost-effective biocatalytic systems.

Ecotoxicity evaluations confirmed that laccases immobilized on biochar completely eliminated the phytotoxicity and antimicrobial inhibition of BG dye. Degraded BG treated with SCBM-TvL stimulated the growth of all four tested biotechnologically important microorganisms, while BG degraded by SCBM-CL stimulated the growth of three out of four microorganisms and exhibited no inhibitory effect on the fourth microorganism, *C. albicans*. Similarly, degraded BG solutions showed no phytotoxic effects, while the SCBM-TvL-treated BG exerted a phytostimulatory effect on wheat seeds, with a GI of 116.58%. In addition, the degradation process resulted in a COD reduction of more than 80%, further confirming the reduction in the organic load and the improved quality of the treated effluent. Overall, the findings highlight acid-modified biochar as an outstanding, cost-effective carrier for laccases, underscoring that enzyme origin influences catalytic performance. Furthermore, this study demonstrates the significant contribution of the air-lift system in providing a scalable and sustainable strategy for wastewater remediation. The work establishes a solid foundation for further research into scaled-up applications, confirming that the biochar–enzyme system holds immense potential as a versatile solution for environmental management.

## Figures and Tables

**Table 1:** Surface area, pore volume and pH_pzc_ of SCB and SCBM.

Material	Surface Area (m^2^/g)	Pore Volume (cm^3^/g)	pH_pzc_
SCB	182.00	0.0744	6.45
SCBM	306.89	0.1298	3.93

**Table 2:** Phytotoxicity parameters for BG solution before and after treatment with SCBM-CL and SCBM-TvL.

Parameters	BG Solution	Degraded BG with SCBM-CL	Degraded BG with SCBM-TvL
RLV [%]	63.73 ± 0.47 ^E^	93.76 ± 0.56 ^C^	124.91 ± 0.74 ^A^
RGV [%]	60.00 ± 0.36 ^F^	93.33 ± 0.45 ^C^	93.33 ± 0.62 ^C^
GI [%]	38.24 ± 0.28 ^G^	87.51 ± 0.41 ^D^	116.58 ± 0.56 ^B^

Values are expressed as mean ± SD (*n* = 3). Different uppercase letters indicate statistically significant differences according to Tukey’s HSD multiple-comparison test (*p* < 0.05), while means sharing at least one letter are not significantly different.

**Table 3:** Effect of BG solution before and after treatment with SCBM-CL and SCBM-TvL.

Microorganism	BG Solution	Degraded BG with SCBM-CL	Degraded BG with SCBM-TvL
*E. coli*	25.17 ± 0.41 ^aC^	7.32 ± 0.22 ^bH^	13.24 ± 0.34 ^bE^
*L. rhamnosus*	33.67 ± 0.52 ^aB^	2.70 ± 0.18 ^bI^	9.13 ± 0.28 ^bG^
*C. albicans*	44.120.61 ^aA^	0 ^J^	8.41 ± 0.25 ^bG^
*S. cerevisiae*	0 ^J^	9.60 ± 0.27 ^bF^	14.45 ± 0.37 ^bD^

^a^ inhibition percentage, ^b^ stimulation percentage. Values are expressed as mean ± SD (*n* = 3). Different uppercase letters indicate statistically significant differences according to Tukey’s HSD multiple-comparison test (*p* < 0.05), while means sharing at least one letter are not significantly different. A value of 0 indicates that no stimulatory or inhibitory effect on microbial growth was observed compared with the corresponding control.

## Data Availability

The original contributions presented in the study are included in the article/Supplementary Materials, and further inquiries can be directed to the corresponding author.

## Supplementary Materials

The following supporting information can be downloaded at https://www.mdpi.com/article/10.3390/polym18202563/s1. Figure S1: Air-lift reactor; Table S1: Characteristics of ALR. Figure S2: EDX of SCBM: before (a) and after CL immobilization (b); Figure S3: Decolorization efficiency as a function of contact time by: SCBM-CL and deactivated SCBM-CL b) SCBM-TvL and deactivated SCBM-TvL in batch system; Table S2: CL activity in reaction and washing supernatants during reuse of SCBM-CL in the batch system; Table S3: TL activity in reaction and washing supernatants during reuse of SCBM-TvL in the batch system; Figure S4: Decolorization efficiency as a function of contact time by: SCBM-CL and deactivated SCBM-CL b) SCBM-TvL and deactivated SCBM-TvL in batch system.
