# Supplementary material for: A Comparative Evaluation of Dye Removal in Batch and Air-Lift Systems by Commercial and Crude Laccase Immobilized on Biochar
Source: Polymers (Basel). 2026 Oct 17;18(20):2563. doi: 10.3390/polym18202563 (PMC13709209; doi:10.3390/polym18202563)
Supplement: Supplementary file 1 [file polymers-18-02563-s001.zip › polymers-4597931-supplementary.pdf]

Supplementary Material

Type of the Paper Article.

A Comparative Evaluation of Dye Removal in Batch and Air-Lift Systems by Commercial and Crude Laccase Immobilized on Biochar

Anja Antanasković [1]*, Milan Milivojević [2], Zorica Lopičić [1], Suzana Dimitrijević-Branković [2], Tamara Vujatović-Velimirov [3], Nevena Ilić [4]

[1] Institute for Technology of Nuclear and Other Mineral Raw Materials, Franchet d'Esperey Boulevard 86, Belgrade, Serbia
[2] Faculty of Technology and Metallurgy, University of Belgrade, Karnegijeva 4, Belgrade, Serbia
[3] Military Technical Institute, Ratka Resanovića 1, Belgrade, Serbia
[4] Inovation Centre of Faculty of Technology and Metallurgy, Karnegijeva 4, Belgrade, Serbia
* Correspondence: a.antanaskovic@itnms.ac.rs

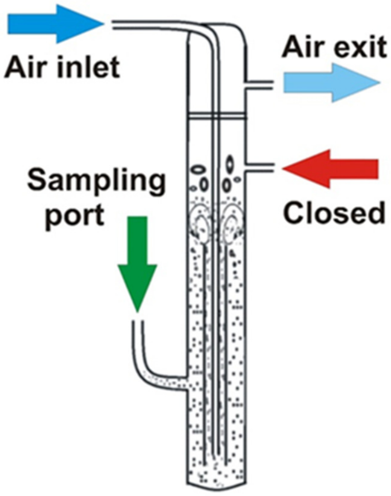

Figure S1. Air-lift reactor

Table S1. Characteristics of ALR

Total/Working volume (L)	0.3/0.150
Total/Liquid height (m)	0.46/0.19
Height or riser draft-tube (m)	0.15
Reactor/riser outer diameter (m)	0.035/0.021
Sparger type	Perforated disc with 8 x 1 mm orifices

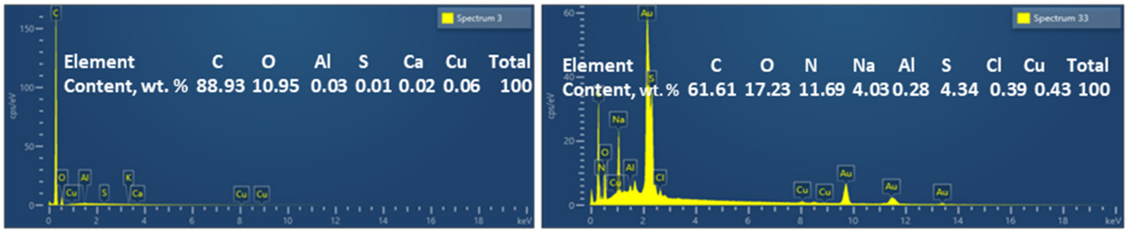

Figure S2. EDX of SCBM: before (a) and after CL immobilization (b)

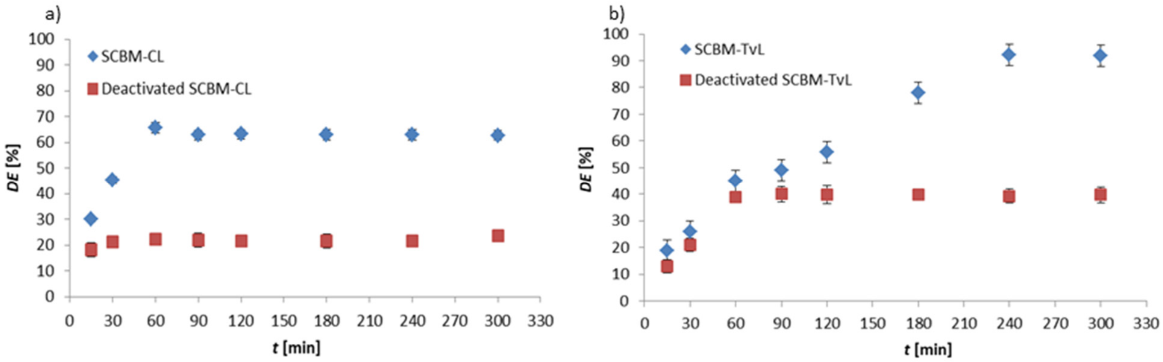

Figure S3. Decolorization efficiency as a function of contact time by: SCBM-CL and deactivated SCBM-CL b) SCBM-TvL and deactivated SCBM-TvL in batch system

Table S2. CL activity in reaction and washing supernatants during reuse of SCBM-CL in the batch system

Cycle	Decolorized supernatant (IU/L)	1st washing supernatant (IU/L)	2nd washing supernatant (IU/L)
1.	4.21	9.56	3.76
2.	6.56	8.71	3.15
3.	7.24	6.48	2.28
4.	8.37	6.13	2.09
5.	9.11	4.92	1.96

Table S3. TL activity in reaction and washing supernatants during reuse of SCBM-TvL in the batch system

Cycle	Decolorized supernatant (IU/L)	1st washing supernatant (IU/L)	2nd washing supernatant (IU/L)
1.	1.81	2.24	1.21
2.	1.54	2.18	1.19
3.	1.62	1.98	1.23
4.	1.64	1.96	1.21
5.	1.58	1.99	1.19
6.	1.53	2.07	1.24
7.	1.78	2.19	1.26
8.	1.86	2.31	1.36
9.	2.24	2.48	2.28
10.	2.76	2.75	2.39
11.	3.38	2.99	2.44

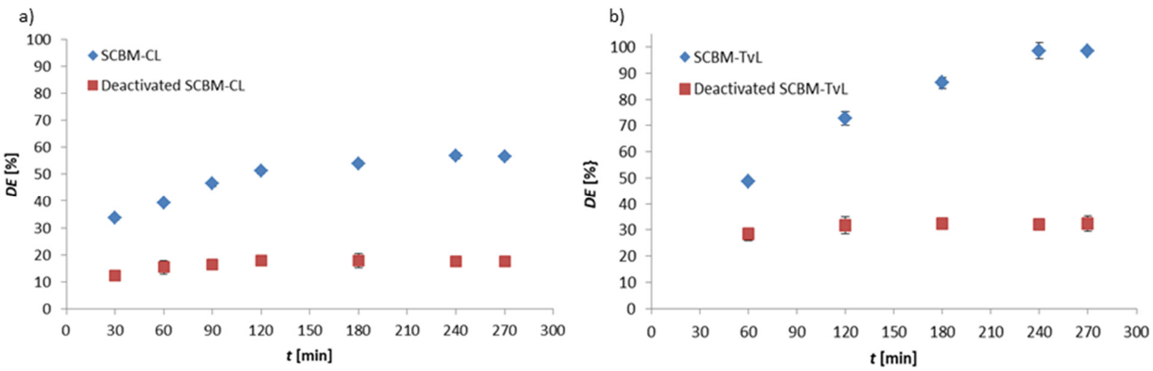

Figure S4. Decolorization efficiency as a function of contact time by: SCBM-CL and deactivated SCBM-CL b) SCBM-TvL and deactivated SCBM-TvL in batch system
